# Label-Free Detection of Cu^2+^ and Hg^2+^ Ions Using Reconstructed Cu^2+^-Specific DNAzyme and G-quadruplex DNAzyme

**DOI:** 10.1371/journal.pone.0073012

**Published:** 2013-09-06

**Authors:** Hui Li, Xiao-Xi Huang, Yang Cai, Hao-Jie Xiao, Qiu-Fen Zhang, De-Ming Kong

**Affiliations:** 1 State Key Laboratory of Medicinal Chemical Biology, Nankai University, Tianjin, P. R. China; 2 Synergetic Innovation Center of Chemical Science and Engineering, Tianjin, P. R. China; CNR, Italy

## Abstract

Label-free metal ion detection methods were developed. To achieve these, a reconstructed Cu^2+^-specific DNA-cleaving DNAzyme (Cu^2+^-specific DNAzyme) with an intramolecular stem-loop structure was used. G-quadruplex-forming G-rich sequence(s), linked at the ends of double-helix stem of an intramolecular stem-loop structure, was partly caged in an intramolecular duplex or formed a split G-quadruplex. Cu^2+^-triggered DNA cleavage at a specific site decreased the stability of the double-helix stem, resulting in the formation or destruction of G-quadruplex DNAzyme that can effectively catalyze the 2,2′-azinobis(3-ethylbenzothiazoline)-6-sulfonic acid (ABTS)-H_2_O_2_ reaction. Based on these, two label-free, cost-effective and simple Cu^2+^ sensors were designed. These two sensors followed different detection modes: ‘turn-on’ and ‘turn-off’. As for the ‘turn-on’ sensor, the intramolecular stem-loop structure ensured a low background signal, and the co-amplification of detection signal by dual DNAzymes (Cu^2+^-specific DNAzyme and G-quadruplex DNAzyme) provided a high sensitivity. This sensor enabled the selective detection of aqueous Cu^2+^ with a detection limit of 3.9 nM. Visual detection was possible. Although the ‘turn-off’ sensor gave lower detection sensitivity than the ‘turn-on’ one, the characteristics of cost-effectiveness and ease of operation made it an important implement to reduce the possibility of pseudo-positive or pseudo-negative results. Combining the ability of Hg^2+^ ion to stabilize T-T base mismatch, above dual DNAzymes-based strategy was further used for Hg^2+^ sensor design. The proposed sensor allowed the specific detection of Hg^2+^ ion with a detection of 4.8 nM. Visual detection was also possible.

## Introduction

With more and more electronic and industrial waste going to the land, rivers and air, environmental pollution has become one of the most important problems in the world. Heavy metal ions in the waste can cause huge damage to human body through food, water and air [1]. In order to avoid the poison of heavy metal to ecosystem, cost-effective and accurate detection of these metal ions is necessary. To date, numerous approaches, such as colorimetric [2], fluorescent [3], atomic absorption/emission spectroscopy [4], inductively coupled plasma mass spectroscopy (ICP-MS) [5] and electrochemical assays [6], have been developed. Most of them require sophisticated equipments or sample treatments [7]. Recently, DNAzymes have emerged as a promising class of candidate to build sensors [8]. As efficiently catalytic DNA molecules, DNAzymes can be superior in terms of stability, cost-effectiveness and ease of modification [9]. Methods based on DNAzymes are extremely important to meet the requirement for simple, specific and accurate detection of heavy metal ions, especially at low concentrations. Several facile fluorescent and colorimetric sensors based on heavy metal ion-specific DNAzymes have been reported [10–17].

Recently, we reconstructed the Cu^2+^-dependent DNAzyme reported by Carmi et al. [18,19], and thus designed a Cu^2+^-sepecific fluorescent sensor [20]. An attractive feature of the reconstructed DNAzyme is that it makes possible the use of an intramolecular stem-loop structure [20]. The fluorophore/quencher pair labeled at the two ends of the stem-loop structure can contact closely, conferring the fluorescent sensor with a low background fluorescence signal and thus high detection sensitivity. However, this fluorescent sensor needs a dual-labeled fluorescent oligonucleotide probe to produce an efficient fluorescence switch for detection. Considering the price and the difficulty of preparing such probes, it is interesting and significant to develop a simple, cost-effective, and label-free method for Cu^2+^ detection.

G-quadruplex DNAzymes are peroxidase-mimicking artificial enzymes formed by unlabelled G-rich oligonucleotides and hemin. Base on target-triggered formation or destruction of G-quadruplexes, a number of label-free colorimetric and/or chemiluminescence detection methods have been reported [21,22]. In this paper, by using G-quadruplex DNAzyme-catalyzed chromogenic reactions indicating the DNA cleavage reaction of the reconstructed Cu^2+^-specific DNAzyme, label-free Cu^2+^ detection, following ‘turn-on’ or ‘turn-off’ mode, was easily achieved. Visual detection was also possible. In addition, combining the stabilizing ability of Hg^2+^ to T-T mismatches, this reconstructed Cu^2+^-specific DNAzyme was further used for label-free detection of Hg^2+^.

## Results and Discussion

### Design of ‘turn-on’ Cu^2+^ sensor

The proposed Cu^2+^ sensor was designed on the basis of two DNAzymes: a Cu^2+^-specific DNA-cleaving enzyme (Cu^2+^-specific DNAzyme) and a G-quadruplex DNAzyme. The used Cu^2+^-specific DNAzyme is a reconstructed version of the original one [19]. This reconstructed DNAzyme also uses two oligonucleotide strands, but one strand (we name it **MB**) exists as a molecular beacon-like intramolecular stem-loop structure, and the cleavage site is embedded in the double-helix stem. The other strand is a polypurine oligonucleotide (**PPO**), it can hybridize with the loop of **MB** to form a DNA triple-helix structure, thus providing a Cu^2+^ ion binding site and leading to the Cu^2+^-specific DNA cleavage reaction at a specific site.

**Figure 1:**
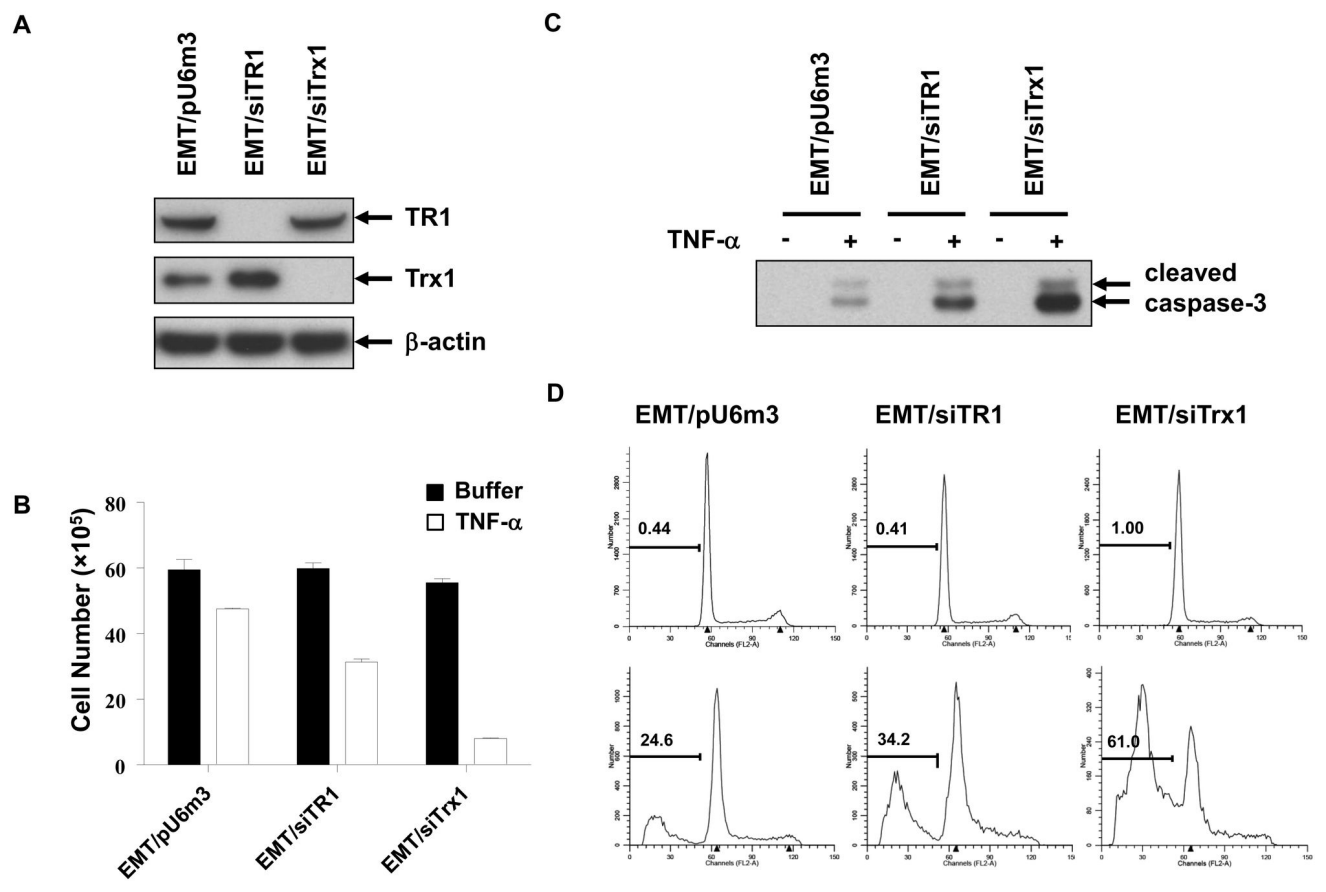
Working mechanism of the dual DNAzymes-based. (a) ‘turn-on’ Cu^2+^ sensor, (b) ‘turn-off’ Cu^2+^ sensor and (e) ‘turn-on’ Hg^2+^ sensor. (c, d) Mechanistic representation of switching catalytic activity of the Cu^2+^-specific DNAzyme by modulating the pocket size. (c) Increasing the pocket size makes the DNAzyme lose catalytic activity. (d) Reducing the pocket size via the formation of double-helix stem recovers the catalytic activity.

To develop a ‘turn-on’ Cu^2+^ sensor, a G-quadruplex-forming G-rich sequence is linked to the 3′-end of the stem of **MB**, and the 5′-end of the stem is appropriately extended by the complementary sequence of the G-rich sequence. The obtained oligonucleotide (we name it **MB-Cu1**, Figure 1a) also adopts an intramolecular stem-loop structure. The G-rich sequence is partially caged in the double-helix stem, not able to form a G-quadruplex. The hybridization of **MB-Cu1** with **PPO** and subsequent Cu^2+^-triggered DNA cleavage convert the stable intramolecular double-helix stem to a less stable intermolecular duplex. The decrease of stability results in the release of G-rich sequence, which folds into a G-quadruplex and assembles into a peroxidase-like DNAzyme in the presence of hemin. The resulting G-quadruplex DNAzyme can effectively catalyze the H_2_O_2_-mediated oxidation of 2,2′-azino-bis(3-ethylbenzothiozoline)-6-sulfonic acid (ABTS) to form the colored radical product ABTS^•+^ [23], which has a maximal absorption at 419 nm. Thus, the change in the absorption signal at this wavelength can be employed to monitor the formation of the G-quadruplex structure, which is closely related to Cu^2+^ concentration.

The as-designed Cu^2+^ sensor (Figure 1) has two important characteristics: one is the use of dual DNAzymes: a Cu^2+^-specific DNAzyme and a G-quadruplex DNAzyme. During Cu^2+^-specific DNA cleavage reaction, Cu^2+^ can be reused. One Cu^2+^ ion can trigger several DNA cleavage reactions, thus achieving the enrichment of G-quadruplex DNAzyme. The resulting G-quadruplex DNAzyme is subsequently used as the catalyst of ABTS-H _2_O_2_ reaction to give enhanced detection signal. Co-amplification of dual DNAzymes can provide the sensing system with high sensitivity. The use of G-quadruplex DNAzyme also makes label-free detection possible.

**Figure 2:**
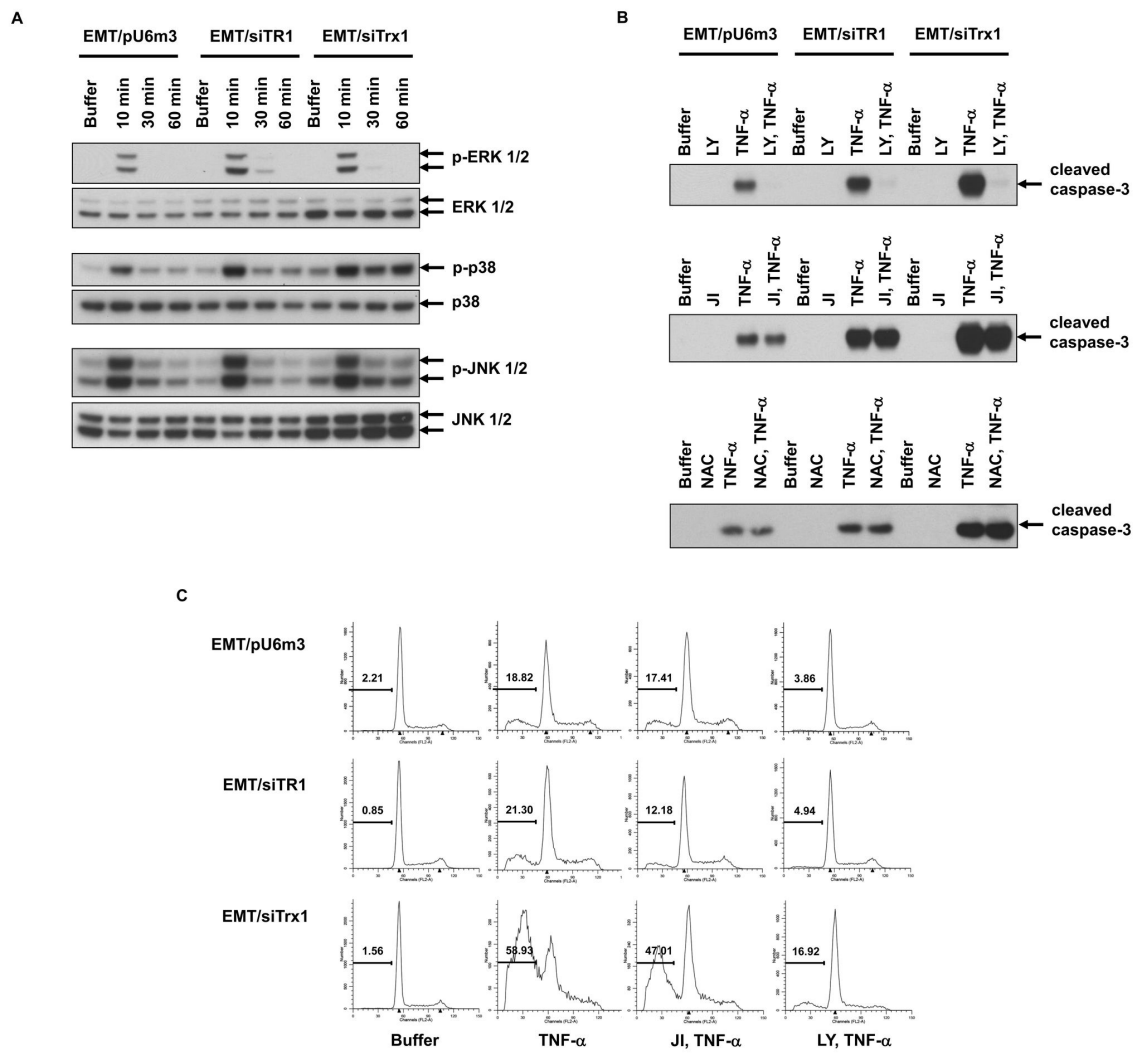
UV–Vis absorption spectra of the designed sensor. (**a**) The absorption signal of the ‘turn-on’ Cu^2+^-sensing system in the absence or presence of 200 nM Cu^2+^. Line 1 represents the sensing system containing free hemin only. The insert shows the photographed images of the reaction tubes. (**b**) The absorption signal of the ‘turn-off’ Cu^2+^-sensing system in the absence or presence of 200 nM Cu^2+^. (**c**) The absorption signal of the sensing system containing two oligonucletodies that form similar structures to the Cu^2+^-specific DNAzyme but with different pocket sizes. (**d**) The absorption signal of the ‘turn-on’ Hg^2+^-sensing system in the absence or presence of 0.5 µM Cu^2+^ and 1 µM Hg^2+^. The insert shows the photographed images of the reaction tubes.

The other characteristic is the use of the reconstructed Cu^2+^-specific DNAzyme. There are three factors that greatly affect the sensitivity of the proposed sensor: blockage of G-quadruplex formation in the absence of Cu^2+^, Cu^2+^-triggered DNA cleavage, and subsequent release of G-rich sequence. The reconstruction of the Cu^2+^-specific DNAzyme does not change the efficiency of DNA cleavage process, but does bring great effects to the first and the third factors. It is well known that an intramolecular duplex is much more stable than the intermolecular one with identical base sequence [24]. For example, a molecular beacon with 5-base pair stem can keep a stable stem-loop structure even at 50-60 ^o^C [25]. That is to say, intramolecular double-helix stem structure of the reconstructed DNAzyme can effectively block the formation of catalytic G-quadruplex, thus keeping the background signal at a low level. In addition, the extraordinary stable intramolecular stem-loop structure makes it possible to use a relatively short stem, thus shortening the length of the intermolecular duplex resulted by DNA cleavage reaction. This makes the release of G-rich sequence much easier. As shown in Figure 2a, in the absence of Cu^2+^, the **MB-Cu1**/**PPO**/hemin system showed a low absorption signal, which was comparable to that caused by inherently catalytic activity of free hemin. However, the addition of 200 nM Cu^2+^ could increase the absorption signal of the sensing system to a much higher level, attributed to the Cu^2+^-triggered DNA cleavage and subsequent formation of the catalytic G-quadruplex DNAzyme as described in Figure 1a.

**Figure 3:**
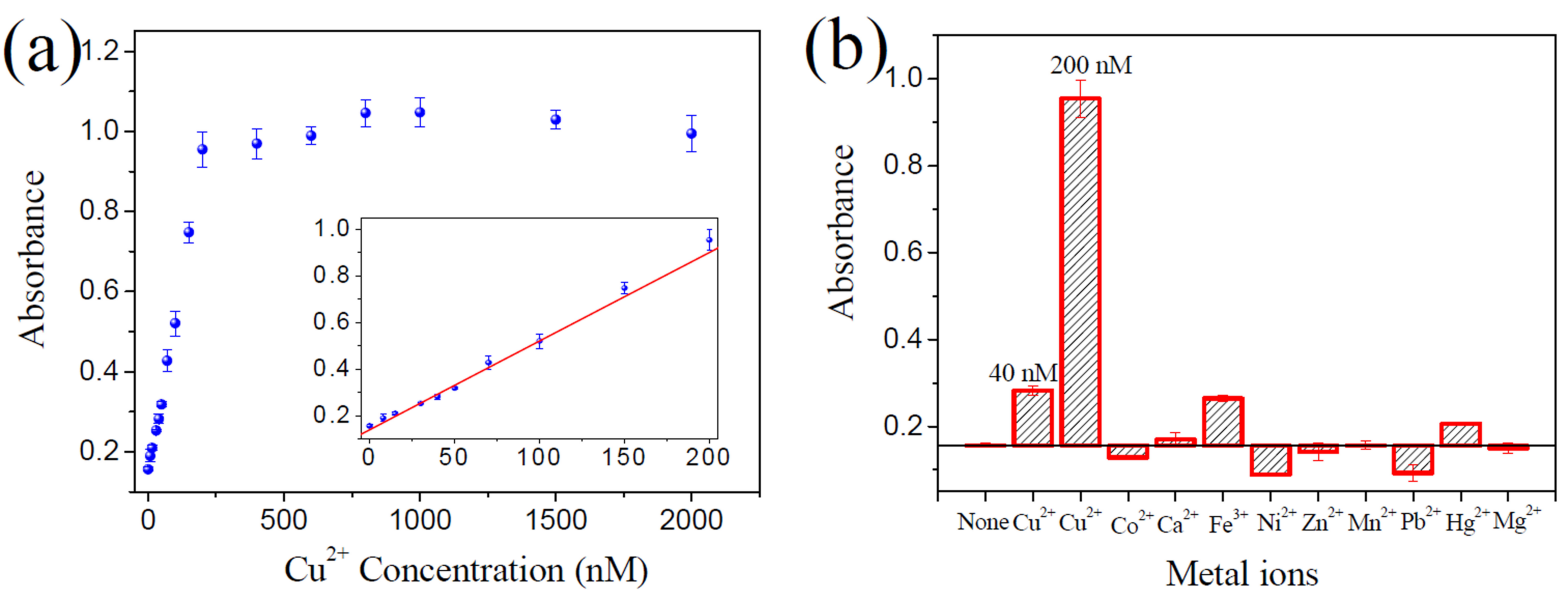
Sensitivity and selectivity of the ‘turn-on’ Cu^2+^ sensor. (a) Absorption signal response of the sensing system to Cu^2+^ concentration. The insert shows the absorption signal change in the Cu^2+^ concentration range of 8-200 nM. The solid line represents a linear fit to the data. (b) Selectivity of the proposed sensor for Cu^2+^ over other competing divalent metal ions. The absorption signal changes of the sensing system induced by different competing metal ions (200 µM) are shown. The concentrations of Cu^2+^ are shown at the top of the bars. All experiments were performed in triplicate.

To study the feasibility of this method for Cu^2+^ quantitation, the absorption signal of the sensing system was measured as a function of Cu^2+^ concentration. As shown in Figure 3a, the absorption signal greatly increased by adding Cu^2+^, and nearly reached a plateau when the Cu^2+^ concentration exceeded 200 nM. A linear relationship (*R* ^2^ = 0.9869) was observed with Cu^2+^concentrations from 8 to 200 nM. On the basis of 3σ/slope of the calibration curve (where σ is the standard deviation of the blank samples), the calculated detection limit was 3.9 nM Cu^2+^, which is much lower the 20 μΜ maximum contamination level set by the US EPA for drinking water, and represents one of the sensitive biosensors of Cu^2+^ with good linearity for quantitative analysis of Cu^2+^ [26–29]. The reproducibility of the proposed method was evaluated by performing ten repetitive experiments for 200 nM Cu^2+^. All of the relative standard deviations (RSD) of intra- (3.12%) and intergroup (2.96%) were <5%, indicating that our assay protocol had good reproducibility. Of note, large absorption signal change could be observed between the systems with or without Cu^2+^, it could even be discriminated by the naked eyes. That is, the proposed ‘turn-on’ sensor can be developed as a simple method for visual detection of Cu^2+^.

To evaluate the selectivity of this ‘turn on’ Cu^2+^-sensing system, nine competing environmentally relevant metal ions, including Co^2+^, Ca^2+^, Fe^3+^, Ni^2+^, Zn^2+^, Mn^2+^, Pb^2+^, Hg^2+^ and Mg^2+^ were assayed. As shown in Figure 3b, among these competing metal ions, only Fe^3+^ and Hg^2+^ caused detectable changes of fluorescence. However, the change of absorbance signal induced by 200 µM Fe^3+^ was similar to, but a little lower than, that induced by 40 nM Cu^2+^, indicating that the sensor selectivity for Cu^2+^ was at least 5000-fold higher than that for Fe^3+^. As for Hg^2+^, the selectivity was much higher.

To investigate whether the method was applicable to real samples, known amounts of Cu^2+^ were added to real water samples and the recovery values were determined from the calibration graph. As shown in Table S1, the recovery of added Cu^2+^ was in the range of 92.2~108.9%, indicating that this method is able to detect Cu^2+^ in real samples with little interference.

### Design of ‘turn-off’ Cu^2+^ sensor

The reconstructed DNAzyme can also be easily used to develop a ‘turn-off’ Cu^2+^ detection method. In this Cu^2+^ sensor, two G-rich sequences, each cannot form a G-quadruplex by itself, are linked to the 5′- and 3′-end of the stem of **MB**, respectively (we name the obtained oligonucleotide as **MB-Cu2**, Figure 1b). With the help of double-helix stem, the two G-rich sequences closely contact to each other, thus folding into a split G-quadruplex [30,31], which can form a catalytic G-quadruplex DNAzyme upon hemin binding. Cu^2+^-triggered DNA cleavage can also result in the dissociation of the split G-quadruplex, leading to the reduction of the catalytic activity and subsequent decrease of the absorbance signal of the sensing system. As shown in Figure 2b, in the absence of Cu^2+^, the **MB-Cu2**/**PPO**/hemin system showed a high catalytic activity towards ABTS-H _2_O_2_ reaction, thus giving a high level of absorption signal. The addition of 200 nM Cu^2+^ led to an obvious decrease of the absorption signal as expected. Of note, a relatively high absorption signal was still observed in the presence of 200 nM Cu^2+^. It is not surprise because the formation of split G-quadruplex at the end of double-helix stem enhances the stability of the stem, thus increasing the difficulty of dissociation of the split G-quadruplex after DNA cleavage.

**Figure 4:**
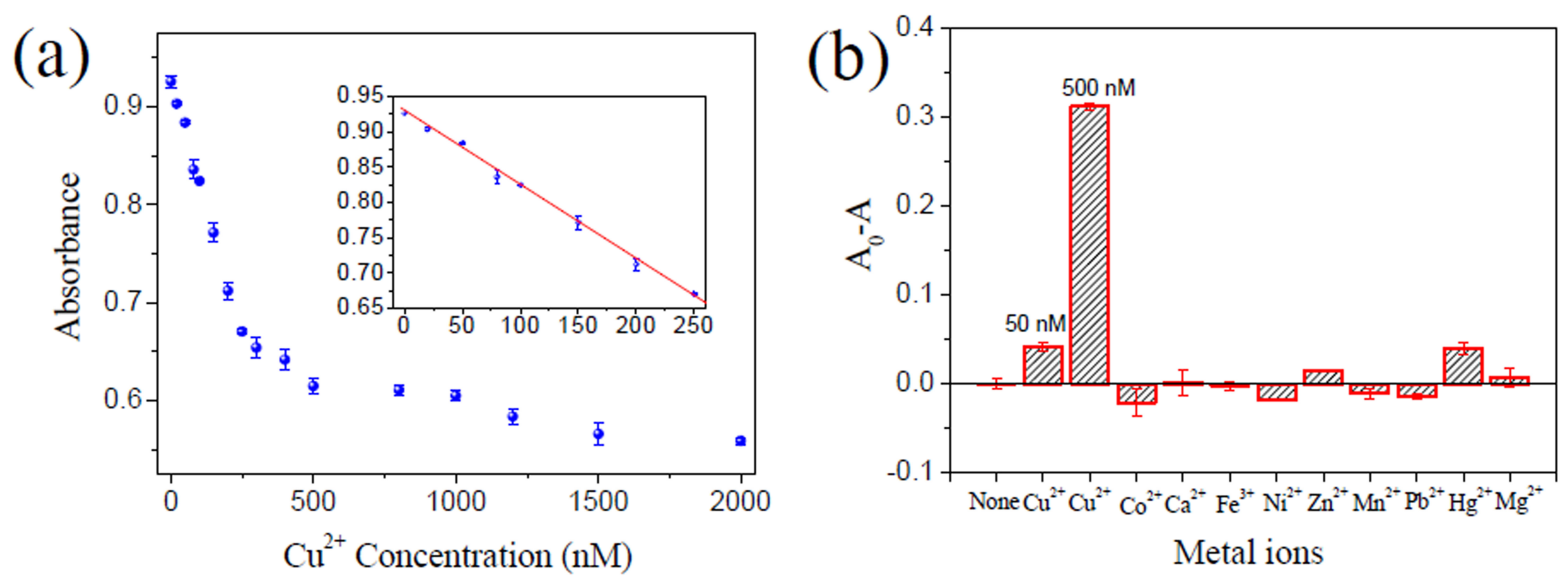
Sensitivity and selectivity of the ‘turn-off’ Cu^2+^ sensor. (**a**) Absorption signal response of the sensing system to Cu^2+^ concentration. The insert shows the absorption signal change in the Cu^2+^ concentration range of 0-250 nM. The solid line represents a linear fit to the data. (**b**) Selectivity of the proposed sensor for Cu^2+^ over other competing divalent metal ions. The absorption signal changes of the sensing system induced by different competing metal ions (5 µM) are shown. The concentrations of Cu^2+^ are shown at the top of the bars. All experiments were performed in triplicate.

The feasibility of this ‘turn-off’ sensor for Cu^2+^ detection was also studied. As shown in Figure 4a, the absorption signal of the sensing system continuously decreased with increasing Cu^2+^ concentration from 0 to 2000 nM, and a good linear relationship (*R* ^2^ = 0.9963) between the absorption signal and Cu^2+^ concentration was observed over a range of 20~250 nM. The calculated detection limit was 16.6 nM, which was higher than that of the ‘turn-on’ method described above. This may be attributed to the high background and large standard deviation of the blank samples, which are the inherent drawbacks often faced by ‘turn-off’ detection methods. Of note, its detection limit is still much lower than the 20 μΜ maximum contamination level set by the US EPA for drinking water. The RSDs of inter- (4.9%) and intragroup (2.1%) were all <5%. This indicates that this ‘turn-off’ detection mode has good reproducibility. This ‘turn-off’ detection method also showed a good selectivity for Cu^2+^ compared to potential competing metal ions (Figure 4b). Among the tested competing metal ions, only Hg^2+^ caused the detectable absorbance signal change, but the signal change caused by 5 µM Hg^2+^ was only similar to that caused by 50 nM Cu^2+^, indicating that the selectivity of the sensor for Cu^2+^ was 100-fold than that for Hg^2+^. Like the ‘turn-on’ sensor, the ‘turn-off’ one can also be used to quantify Cu^2+^ in practice. The calculated recovery was in the range of 97.2~101.3% (Table S2). Though the sensitivity and selectivity of the ‘turn-off’ sensor are all not so good as those of the ‘turn-on’ one, the ‘turn-off’ sensor can be used as an important implement for the ‘turn-on’ one. Single detection mode (‘turn-on’ or ‘turn-off’) may suffer from pseudo-positive or pseudo-negative problem. The two label-free detection methods proposed in this work offer the same advantages of cost-effectiveness and ease of operation. Combined use of them is feasible to reduce the possibility of pseudo-positive and pseudo-negative results.

### Design of ‘turn-on’ Hg^2+^ sensor

The experiments mentioned above demonstrated that the reconstructed Cu^2+^-specific DNAzyme provided a good choice for the design of Cu^2+^ sensors. Then, we wonder whether it can be extended to the design of other sensors. As shown in Figure 1, in the complex formed by **MB** and **PPO**, there is a single-stranded loop structure, which likes a pocket. Cu^2+^ can bind into this pocket and trigger DNA cleavage reaction. The size of pocket is specific for Cu^2+^. If several nucleotides are inserted into the loop sequence to enlarge the pocket size, the Cu^2+^-specific DNA cleavage will be almost inhibited completely (Figure 1c), which can be reflected by the low absorption signal of corresponding ABTS-H _2_O_2_ system (Figure 2c). But we found that if the inserted sequence was self-complementary to form a double-helix stem as in the **MB-Hg2/PPO** complex shown in Figure 1d, the pocket size would nearly recover. As a result, the efficiency of Cu^2+^-specific DNA cleavage also recovered and high absorption signal could be observed in ABTS-H _2_O_2_ system (Figure 2c). This finding provides the possibility for the design of sensors for metal ions that can promote the formation of metal-base pairs [32], and one example is Hg^2+^. It is well known that Hg^2+^ can stabilize T-T base mismatch [33–35]. When the self-complementary stem-forming bases inserted in **MB-Hg2** are replaced by T bases (we name the new oligonucleotide as **MB-Hg1**), the pocket size is enlarged and Cu^2+^-specific DNA cleavage is inhibited. In the presence of Hg^2+^, the pocket size is recovered by the formation of the double-stranded stem containing T-Hg^2+^-T base pairs, thus making Cu^2+^-sepcific DNA cleavage possible (Figure 1e). On the basis of switching modulation of Cu^2+^-specific DNA cleavage by Hg^2+^, a Hg^2+^-specific sensor can be designed.

**Figure 5:**
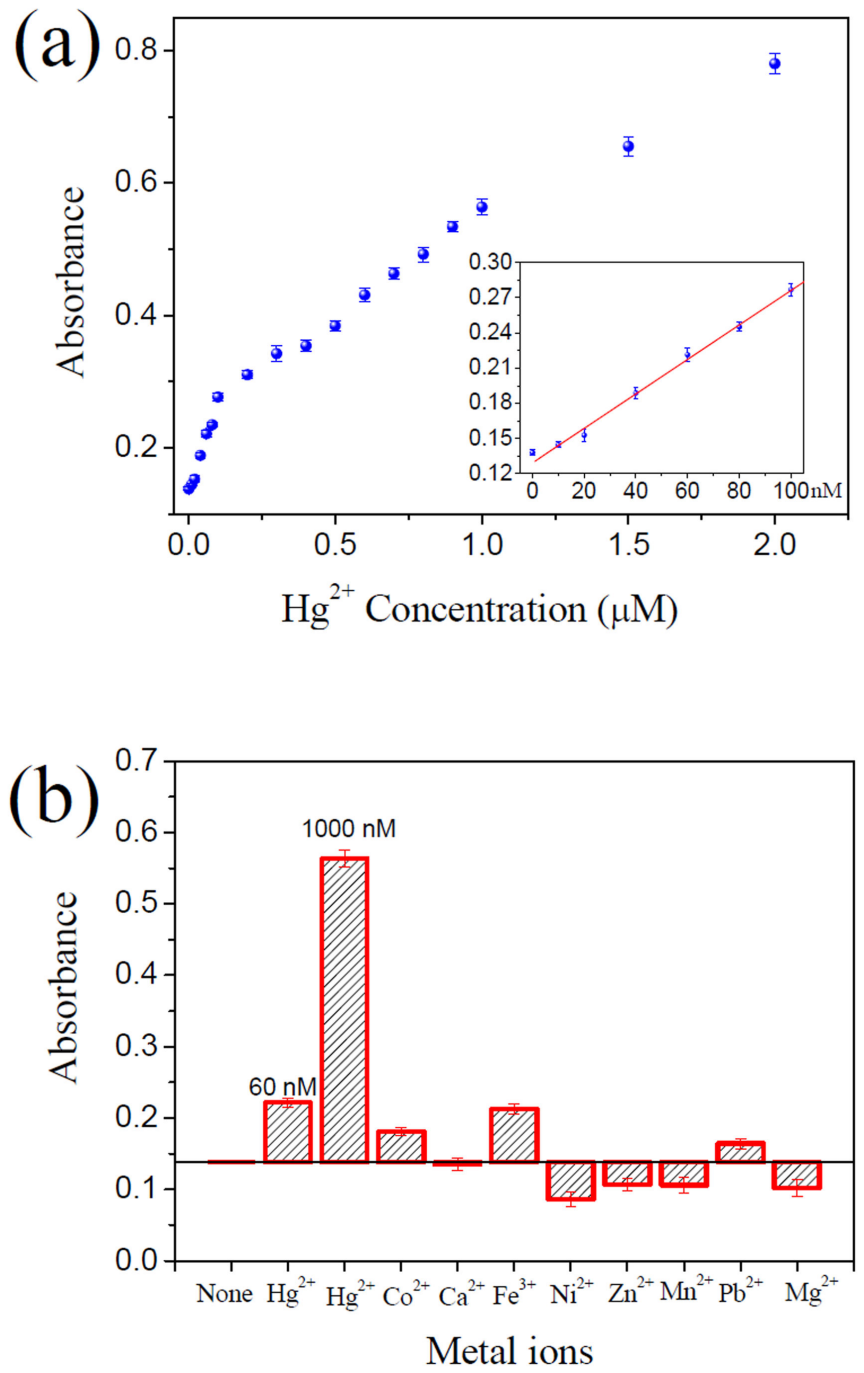
Sensitivity and selectivity of the ‘turn-on’ Hg^2+^ sensor. (**a**) Absorption signal response of the sensing system to Hg^2+^ concentration. The insert shows the absorption signal change in the Cu^2+^ concentration range of 10-100 nM. The solid line represents a linear fit to the data. (**b**) Selectivity of the proposed sensor for Hg^2+^ over other competing divalent metal ions. The absorption signal changes of the sensing system induced by different competing metal ions (20 µM) are shown. The concentrations of Hg^2+^ are shown at the top of the bars. All experiments were performed in triplicate.

The results in Figure 2d demonstrated the feasibility of the proposed Hg^2+^ sensor. In the absence of Hg^2+^, the MB–Hg1/PPO/hemin system containing 500 nM Cu^2+^ showed a very low absorption signal, which was comparable to that of the similar system but without Cu^2+^. This result demonstrated that the efficiency of Cu^2+^-specific DNA cleavage was indeed greatly inhibited by enlarging the pocket size. In the presence of 1 µM Hg^2+^, however, the absorption signal of the sensing system increased to a much high level, which could be attributed to the recovery of the pocket size and re-formation of Cu^2+^-specific DNA-cleaving DNAzyme. A linear relationship (*R* ^2^ = 0.9978) was observed with increasing Hg^2+^ concentrations from 10 to 100 nM. The calculated detection limit was 4.8 nM (Figure 5a), which was also lower than the 10 nM maximum contamination level set by the US EPA for drinking water. Among the tested competing metal ions, Fe^3+^ caused the largest absorbance signal enhancement (Figure 5b), but the signal enhancement caused by 20 µM Fe^3+^ was even lower than that caused by 60 nM Hg^2+^, indicating that the selectivity of the sensor for Hg^2+^ was at least 333-fold greater than that for Fe^3+^. As for other metal ions, the selectivity was much higher. Recovery experiments of water samples (the recovery was in the range 95.5-110.5%) suggested that the Hg^2+^ sensor was able to detect low concentrations of Hg^2+^ in real samples (Table S3).

## Conclusion

In summary, on the basis of G-quadruplex DNAzyme and a reconstructed Cu^2+^-specific DNAzyme, two label-free, cost-effective and simple Cu^2+^ sensors were designed. These two sensors followed different detection modes: ‘turn-on’ and ‘turn-off’. The ‘turn-on’ sensor enabled the selective detection of aqueous Cu^2+^ with a detection limit of 3.9 nM and a linear range of 8-200 nM. Visual detection was possible. The ‘turn-off’ one could be used as an important implement to reduce the possibility of pseudo-positive or pseudo-negative results. Combining the modulation of the pocket size in the Cu^2+^-specific DNAzyme and the switching of Cu^2+^-triggered DNA cleavage by the formation of T-Hg^2+^-T base mismatch, above dual DNAzymes-based strategy was further used for Hg^2+^ sensor design. The proposed sensor allowed the specific detection of Hg^2+^ ion with a detection of 4.8 nM. Visual detection was also possible.

## Materials and Methods

### Materials and reagents

All oligonucleotides (Table 1) were purchased from Sangon Biotech. Co. Ltd (Shanghai, China). The concentrations of the oligonucleotides were represented as single-stranded concentrations. Single-stranded concentrations were determined by measuring the absorbance at 260 nm. Molar extinction coefficients were determined using a nearest neighbour approximation. H_2_O_2_, 2,2′-azinobis(3-ethylbenzothiazoline)-6-sulfonic acid (ABTS), 4-(2-hydroxyethyl)-1- piperazineethanesulfonic acid (HEPES), Triton X-100, L-ascorbic acid and the used metal salts (NaCl, KAc, Hg(Ac)_2_ , Mg(NO_3_)_2_, Cu(NO_3_)_2_, Mn(Ac)_2_, Zn(Ac)_2_, Pb(NO_3_)_2_, Ni(NO_3_)_2_, Co(Ac)_2_ , Fe(NO_3_)_3_ and Ca (Ac)_2_) were obtained from Sigma. All chemical reagents were of analytical grade and used without further puriﬁcation.

**Table 1:** The oligonucleotides used in this work.

**Oligonucleotide strand**	**Sequence^a^**
**MB-Cu1**	5′- C T A C C C A G C CTGGGCCTCTTTCTTCTTTTAGCTTCTTCTTTCTAATACG G C T G G G T A GGGCGGGTTGGG
**MB-Cu2**	5′-GGGTGGGTTA G C CTGGGCCTCTTTCTTCTTTTAGCTTCTTCTTTCTAATACG G C TTTGGGTGGG
**MB-Hg1**	5′- C T A C C C A G C CTGGGTTTCTTGTTTCCTCTTTCTTCTTTTAGCTTCTTCTTTCTAATACG G C T G G G T A GGGCGGGTTGGG
**MB-Hg2**	5′- C T A C C C A G C CTGGGT T C GAAC G A ACCTCTTTCTTCTTTTAGCTTCTTCTTTCTAATACG G C T G G G T A GGGCGGGTTGGG
**PPO**	5′-AAGAAGAAAGAAC

a The italic sequences represent the G-quadruplex-forming sequences; the underlined sequences represent the double-helix stem-forming sequences.

### ‘Turn-on’ colorimetric detection of Cu^2+^

The mixture of **MB-Cu1** (0.5 µM) and **PPO** (1 µM) was prepared in 25 mM HEPES buffer (pH 7.0) containing 300 mM NaCl, 5 mM Mg(NO_3_)_2_ and 5 mM KAc. The mixture was heated to 80 °C for 2 min to remove any possible aggregates, then cooled slowly to 20 °C, and incubated at 20 °C for 60 min to form a stable triplex-helix structure. Subsequently, 2 µM L-ascorbic acid and different concentrations of Cu^2+^ were added to the mixture. After a quick vortex, the mixture was incubated at 20 °C for another 30 min. Then hemin (1 µM, final concentration) was added to the mixture. The mixture was held for 1 h at 20 °C. Then, ABTS (3.2 mM, final concentration) and H_2_O_2_ (2.2 mM, final concentration) were added. The final volume of the mixture was 100 µL. The absorption spectrum of the reaction product ABTS^•+^ was recorded by a TU-1901 UV–vis spectrophotometer after the reaction had run for 4 min. The absorbance at 419 nm was used for quantitative analysis.

### ‘Turn-off’ colorimetric detection of Cu^2+^

The mixture of **MB-Cu2** (0.2 µM) and **PPO** (0.4 µM) was prepared in 25 mM HEPES buffer (pH 7.0) containing 300 mM NaCl. The DNA solution was heated to 80 °C for 2 min, then cooled slowly to 20 °C, and incubated at 20 °C for 60 min to form a stable triplex-helix structure. Then, the other experimental conditions are the same as above.

### ‘Turn-on’ colorimetric detection of Hg^2+^

The mixture of **MB-Hg1** (0.1 µM), **PPO** (0.2 µM) and Hg^2+^ (different concentration) was prepared in 25 mM HEPES buffer (pH 6.6) containing 200 mM NaAc, 5 mM Mg(NO_3_)_2_ and 5 mM KAc. The DNA solution was heated to 80 °C for 2 min, then cooled slowly to 20 °C, and incubated at 20 °C for 60 min to form a stable triplex-helix structure. Subsequently, 2 μM ascorbic acid and 2 µM Cu^2+^ were added to the mixture. Then, the other experimental conditions are the same as above.

## Supporting Information

Table S1 Cu^2+^ recoveries determined by the ‘turn-on’ Cu^2+^ sensor. (DOC)

Table S2 Cu^2+^ recoveries determined by the ‘turn-off’ Cu^2+^ sensor. (DOC)

Table S3 Hg^2+^ recoveries determined by the ‘turn-on’ Hg^2+^ sensor. (DOC)
