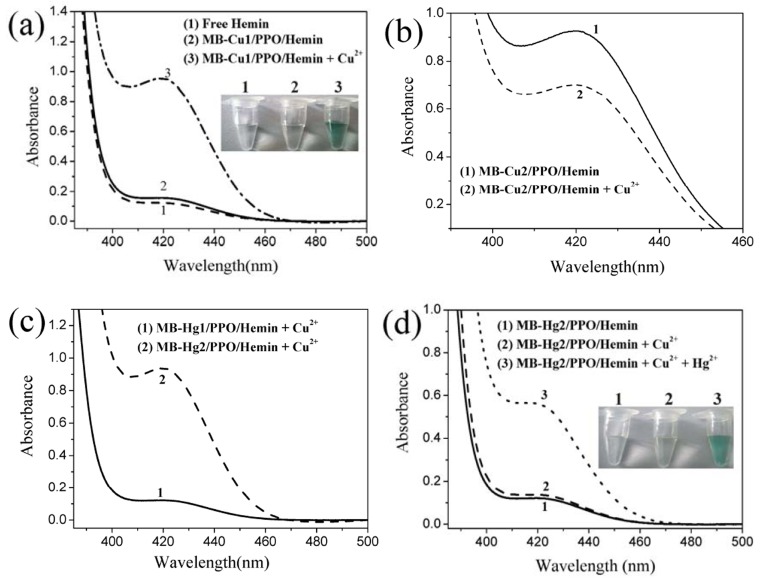# Correction: Label-Free Detection of Cu^2+^ and Hg^2+^ Ions Using Reconstructed Cu^2+^-Specific DNAzyme and G-quadruplex DNAzyme

**DOI:** 10.1371/annotation/57871216-6f4e-4b63-98aa-a5f56c59bc2e

**Published:** 2013-10-07

**Authors:** Hui Li, Xiao-Xi Huang, Yang Cai, Hao-Jie Xiao, Qiu-Fen Zhang, De-Ming Kong

As a result of errors in the typesetting process, the versions of Figures 1 and 2 in the article are incorrect. The correct versions of the articles are available below.

Figure 1: 

**Figure:**
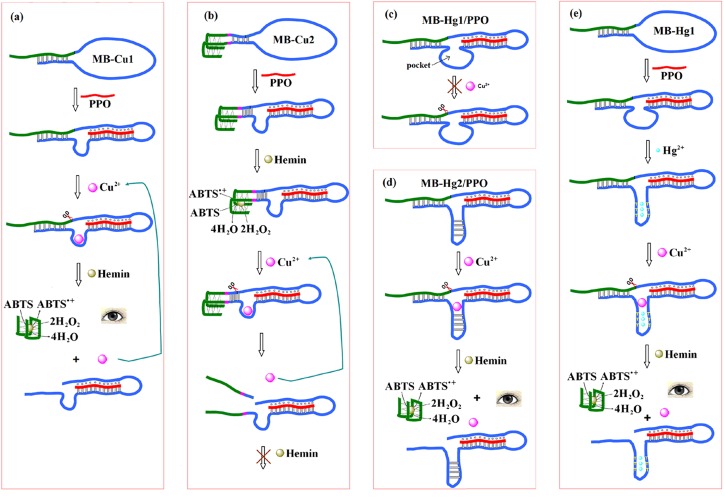


Figure 2: 

**Figure:**