# Supplementary material for: Label-Free Detection of Cu2+ and Hg2+ Ions Using Reconstructed Cu2+-Specific DNAzyme and G-quadruplex DNAzyme
Source: PLoS One. 2013 Sep 6;8(9):e73012. doi: 10.1371/journal.pone.0073012 (PMC3765245; doi:10.1371/journal.pone.0073012)
Supplement: Table S1 — (DOC) [file pone.0073012.s001.doc]

**Table S1. Cu^2+^ recoveries determined by the ‘turn-on’ Cu^2+^** sensor

| **Samples** | **Cu^2+^(nM)** | | | | **Recovery(%)** |
| --- | --- | --- | --- | --- | --- |
|  | **Added** | **Recovered** | | |  |
| Purified water | 100.0 | 95.4 | 104.5 | 97.7 | 92.2 ± 4.7 |
|  | 200.0 | 222.6 | 214.8 | 205.4 | 107.1 ± 4.3 |
| Spring water | 100.0 | 94.3 | 102.3 | 100.2 | 98.9 ± 4.1 |
|  | 200.0 | 198.8 | 217.4 | 217.8 | 105.6 ± 5.4 |
| Tap water | 100.0 | 96.7 | 103.7 | 103.9 | 101.5 ± 4.1 |
|  | 200.0 | 210.6 | 203.8 | 198.9 | 102.2 ± 2.9 |
| Lake water | 100.0 | 99.7 | 108.5 | 104.3 | 104.2 ± 4.4 |
|  | 200.0 | 225.2 | 224.4 | 203.6 | 108.9 ± 6.1 |
