# Supplementary material for: Label-Free Detection of Cu2+ and Hg2+ Ions Using Reconstructed Cu2+-Specific DNAzyme and G-quadruplex DNAzyme
Source: PLoS One. 2013 Sep 6;8(9):e73012. doi: 10.1371/journal.pone.0073012 (PMC3765245; doi:10.1371/journal.pone.0073012)
Supplement: Table S2 — (DOC) [file pone.0073012.s002.doc]

**Table S2. Cu^2+^ recoveries determined by the ‘turn-off’ Cu^2+^** sensor

| **Sample** | **Cu^2+^(nM)** | | | | **Recovery(%)** |
| --- | --- | --- | --- | --- | --- |
|  | **Added** | **Recovered** | | |  |
| Purified water | 150.0 | 157.8 | 147.6 | 150.3 | 101.3±3.5 |
|  | 250.0 | 236.2 | 270.5 | 251.4 | 101.1±6.7 |
| Spring water | 150.0 | 151.5 | 149.4 | 151.9 | 100.6±0.9 |
|  | 250.0 | 246.8 | 237.4 | 254.1 | 98.4±3.3 |
| Tap water | 150.0 | 148.5 | 149.9 | 154.6 | 100.7±2.1 |
|  | 250.0 | 238.0 | 239.2 | 251.4 | 97.2±3.0 |
| Lake water | 150.0 | 143.8 | 144.7 | 150.8 | 97.6±2.5 |
|  | 250.0 | 242.0 | 232.6 | 256.5 | 97.5±4.8 |
