# Supplementary material for: Label-Free Detection of Cu2+ and Hg2+ Ions Using Reconstructed Cu2+-Specific DNAzyme and G-quadruplex DNAzyme
Source: PLoS One. 2013 Sep 6;8(9):e73012. doi: 10.1371/journal.pone.0073012 (PMC3765245; doi:10.1371/journal.pone.0073012)
Supplement: Table S3 — (DOC) [file pone.0073012.s003.doc]

**Table S3. Hg^2+^ recoveries determined by the ‘turn-on’ Hg^2+^** sensor

| **Samples** | **Hg ^2+^(nM)** | | | | **Recovery(%)** |
| --- | --- | --- | --- | --- | --- |
|  | **Added** | **Recovered** | | |  |
| Purified water | 50.0 | 52.2 | 49.9 | 48.1 | 100.2 ± 4.2 |
|  | 100.0 | 100.3 | 99.9 | 96.4 | 99.9 ± 0.4 |
| Spring water | 50.0 | 49.6 | 45.2 | 48.6 | 95.8 ± 4.7 |
|  | 100.0 | 97.4 | 105.2 | 98.4 | 100.3 ± 4.2 |
| Tap water | 50.0 | 52.2 | 47.2 | 53.4 | 97.4 ± 4.2 |
|  | 100.0 | 96.7 | 90.4 | 97.4 | 95.5 ± 4.7 |
| Lake water | 50.0 | 52.7 | 57.4 | 55.7 | 110.5 ± 4.8 |
|  | 100.0 | 94.7 | 95.6 | 118.4 | 102.9 ± 13.4 |
